# Influence of COVID-19 for delaying the diagnosis and treatment of pulmonary tuberculosis–Tianjin, China

**DOI:** 10.3389/fpubh.2022.937844

**Published:** 2022-12-02

**Authors:** Guoqin Zhang, Yanming Yu, Wenqian Zhang, Jian Shang, Shengyu Chen, Xuewen Pang, John E. Oeltmann, Patrick K. Moonan, Mingting Chen, Fan Zhang

**Affiliations:** ^1^Tianjin Center for Tuberculosis Control, Tianjin, China; ^2^US Centers for Disease Control and Prevention, COVID-19 Response, Atlanta, GA, United States; ^3^Chinese Center for Disease Control and Prevention, Beijing, China

**Keywords:** COVID-19, tuberculosis, impact, healthcare seeking, treatment

## Abstract

**Background:**

The COVID-19 pandemic has disrupted the diagnosis, treatment, and care for tuberculosis (TB). Delays in seeking TB care may result in increased community transmission and unfavorable treatment outcomes. We sought to understand the influence of the COVID-19 pandemic on the proportion of patients with TB who delayed seeking the diagnosis and care for TB and explore the reasons for their postponement.

**Methods:**

We surveyed a representative sample of outpatients treated for pulmonary TB from June to November 2020 using an anonymous standardized questionnaire. Multivariable logistic regression was used to calculate adjusted odds ratios (aOR) and 95% confidence intervals (CIs) of factors associated with the postponement of TB care. We used routinely collected surveillance data to assess trends of TB reports before and after the emergence of COVID-19 (2017–2019 vs. 2020–2022) in Tianjin, China.

**Results:**

Among 358 participants who were diagnosed with pulmonary TB during the COVID-19 response, 61 (17%) postponed seeking TB diagnosis due to COVID-19, with 39 (64%) citing fear as the primary reason. Female sex (aOR:2.0; 95% CI: 1.1–3.7), previous antituberculosis treatment (aOR:3.2; 95%CI: 1.4–7.6), and TB diagnosis during the first-level response (aOR = 3.2, 1.7–6.2) were associated with the postponement. Among all 518 participants receiving antituberculosis treatment, 57 (11%) had postponed their regular healthcare visits due to COVID-19, 175 (34%) received no treatment supervision, and 32 (6%) experienced treatment interruption. Compared to 2017–2019, reported pulmonary TB declined by 36.8% during the first-level response to COVID-19, 23.5% during the second-level response, 14% during the third-level response in 2020, and 4.3% in 2021.

**Conclusion:**

The COVID-19 response reduced the number of people who sought and received diagnosis, treatment, and care for TB in Tianjin, China. Integrative programs to ensure access and continuity of TB services should be considered and dual testing for SARS-CoV-2 and *M. tuberculosis* may facilitate finding cases.

## Introduction

The novel coronavirus disease 2019 (COVID-19) was first reported in December 2019 in Wuhan, China, and rapidly spread across the world, resulting in more than 600 million cases and over six million COVID-19-related deaths (1). To contain the COVID-19 epidemic, all provincial administrations in mainland China initiated the response to the public health emergency in 2020, including restriction of public transportation, prohibition of public gatherings, community containment, active contact tracing, and testing (2–4). These interventions played a substantial role in mitigating COVID-19; however, indirectly disrupted access to the diagnosis, treatment, and care for other diseases, such as cancer diagnosis and management in Slovenia (5), emergency healthcare in the Netherlands (6), pediatric healthcare in Italy (7), maternal healthcare in India (8), as well as tuberculosis (TB) in low- and middle-income countries (9, 10). Despite mostly being curable, TB remains a disease of public health importance (11). Globally, COVID-19 public health interventions were expected to heavily impact TB prevention and care (10, 12, 13). In China, individuals with laboratory-confirmed COVID-19 receive compulsory care at designated hospitals, many of which were directly repurposed from TB-designated hospitals. In 2020, China reported the second highest burden of TB in the world (11), yet a substantial reduction in the number of TB cases experienced during the COVID-19 response (14–17). As COVID-19 continues to spread all over the world, the emergence of variants (such as Delta and Omicron variants) complicates response efforts; on the other hand, the interventions against COVID-19 such as active testing, social distancing, and wearing mask provide opportunity as well as challenge for TB control (18); it is important to understand the influence of COVID-19 activities on the implementation of TB services and to inspire new strategies for establishing and sustaining access and continuation of TB care.

Tianjin is one of the four municipalities under the direction of the central government in China, with approximately 14 million permanent residents. Since 2020, in response to the COVID-19 epidemic, the municipal TB-designated hospital, which typically cares for 75% of all patients with TB in the city, was repurposed as a centralized COVID-19 designated hospital; thus, TB services were suspended. Implementation of China CDC's “Dynamic Zero Policy” against COVID-19 resulted in no new locally transmitted COVID-19 cases in 2021 (14), offering an opportunity to evaluate the influence of COVID-19 on access to TB services. We sought to understand the influence of COVID-19 on trends of TB reports, determine the proportion of patients with TB who delayed seeking TB diagnosis and care, and explored the reasons for their postponement during the COVID-19 response in Tianjin, China.

## Materials and methods

### Study design

This is a cross-sectional study, with data from two sources: (1) preplanned surveys in all 16 districts in Tianjin from June to November 2020, including a standardized questionnaire survey collected from a representative sample of pulmonary TB patients, open questionnaire surveys to all TB clinics, and selected non-TB hospitals that reported presumable patients with TB and (2) routinely collected surveillance data of pulmonary TB maintained in TB Information and Management System (TBIMS) in Tianjin during 2017–2022.

### Data collection

**Figure 1:**
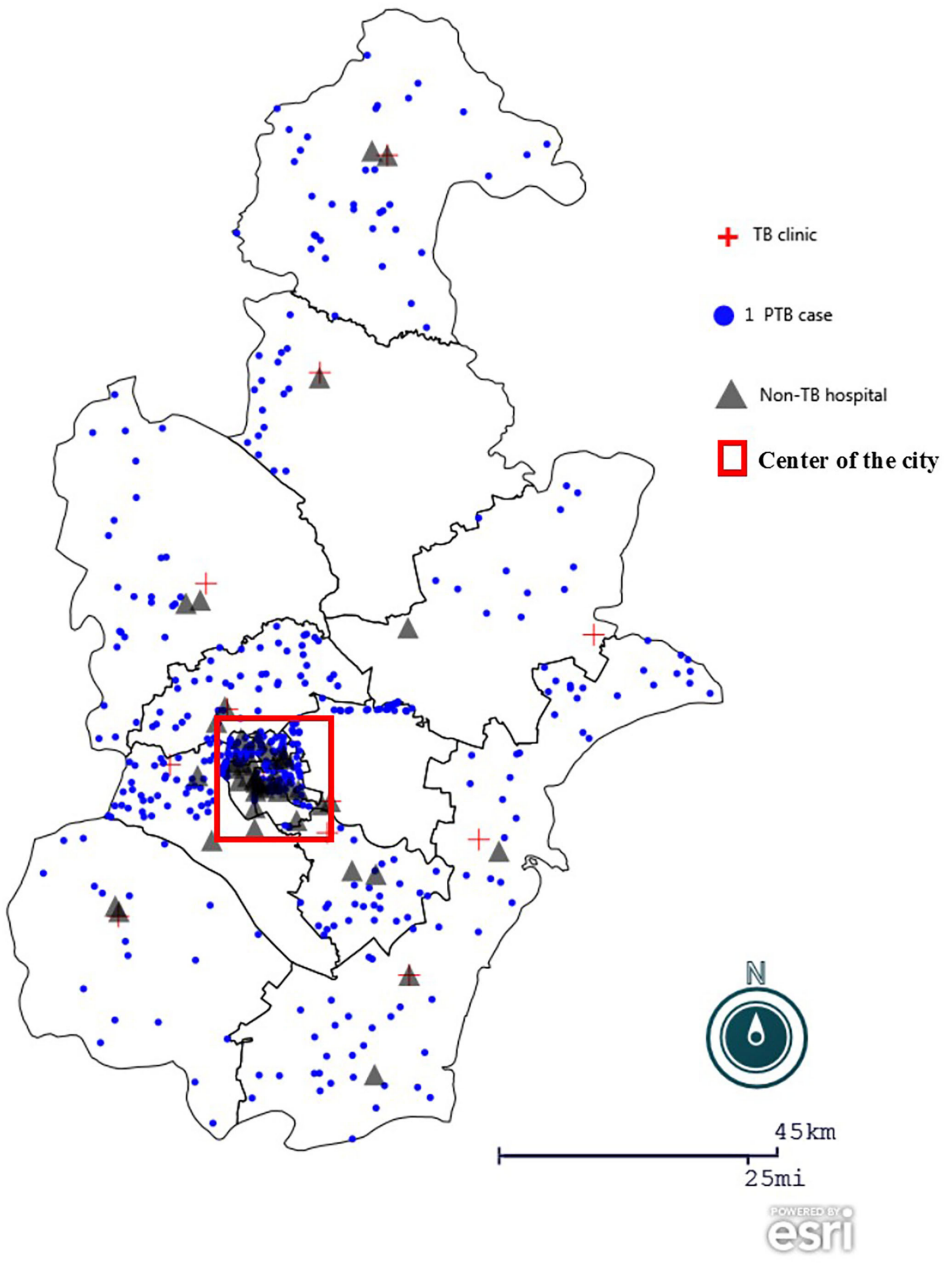
Geographic distribution of participants in the study. The geographic map of Tianjin, China was created using Epi Info (https://www.cdc.gov/epiinfo), with a dot density map to demonstrate district distribution of patient participants, overlaying a spot map to mark TB clinics, non-TB hospitals surveyed. 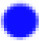: dot density by district according to home address of patients participated in the anonymous questionnaire. 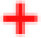: the 12 TB clinics or designated hospital in the city. 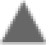: the 45 non-TB hospitals were purposively selected in city and district levels, considering their report of presumable patients with TB in the previous years. 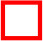: highlight the center of the city, which accounts for 1.4% of the city area and accommodates around one third of the city population.

Consented participants were representatively enrolled according to the criteria (Table 1) at all 11 TB clinics which still provided TB service during the COVID-19 response in Tianjin (Figure 1). We determined the required sample size as ≥384 patients to satisfy a cross-sectional design (*P*_0_ = 50%, δ = 5%) using EpiInfo^TM^ (CDC, Atlanta USA, https://www.cdc.gov/epiinfo). An online (Wenjuanxing, https://www.wjx.cn/) anonymous questionnaire with must-answer and a logic check was developed; self-administered using a mobile phone, with the assistance of medical staff or nurses if participants need it. Consent was obtained orally by medical staff or nurses, as well as written in the questionnaire. The questionnaire included clinical (i.e., symptoms, date of diagnosis, and healthcare-seeking behaviors during the COVID-19 response), demographic (i.e., age, sex, education, residency status, and travel time to TB services), and TB treatment variables (i.e., history of previous antituberculosis treatment and treatment supervision).

**Table 1:** Inclusion and exclusion of study subjects in the study.

**Data source**	**Inclusion**	**Exclusion**
1. Questionnaire survey to pulmonary TB patients	1) At least aged 14 years or more; 2) Diagnosed with pulmonary TB, and receiving antituberculosis treatment; 3) Consent to take part in.	1) Age 13 years or less; 2) With non-pulmonary forms of TB; 3) Refused consent of enrolment.
2. Survey to TB clinics	All TB clinics in the city	NA
3. Survey to non-TB hospitals	1. Non-TB designated hospitals; 2. Report and refer presumable TB	Never report presumable TB in the past years
4. Pulmonary TB surveillance	Pulmonary TB reported during 2017–2022 from TBIMS	Non-pulmonary forms of TB

An open questionnaire survey was conducted in all TB clinics in the city, mainly on TB services provided during the COVID-19 response. We also purposefully selected 45 non-TB hospitals at the city and district levels that reported the majority of presumable patients with TB, to inquire about finding TB cases and referrals.

We exported data on pulmonary TB reported during 2017–2022 from TBIMS without identifiable information such as name, address, and telephone number. The data include information on demography, TB diagnosis, follow-up examination, and treatment outcome, which were collected by physicians in medical charts, and entered within 24 h after TB diagnosis.

### Key time points

**Figure 2:**
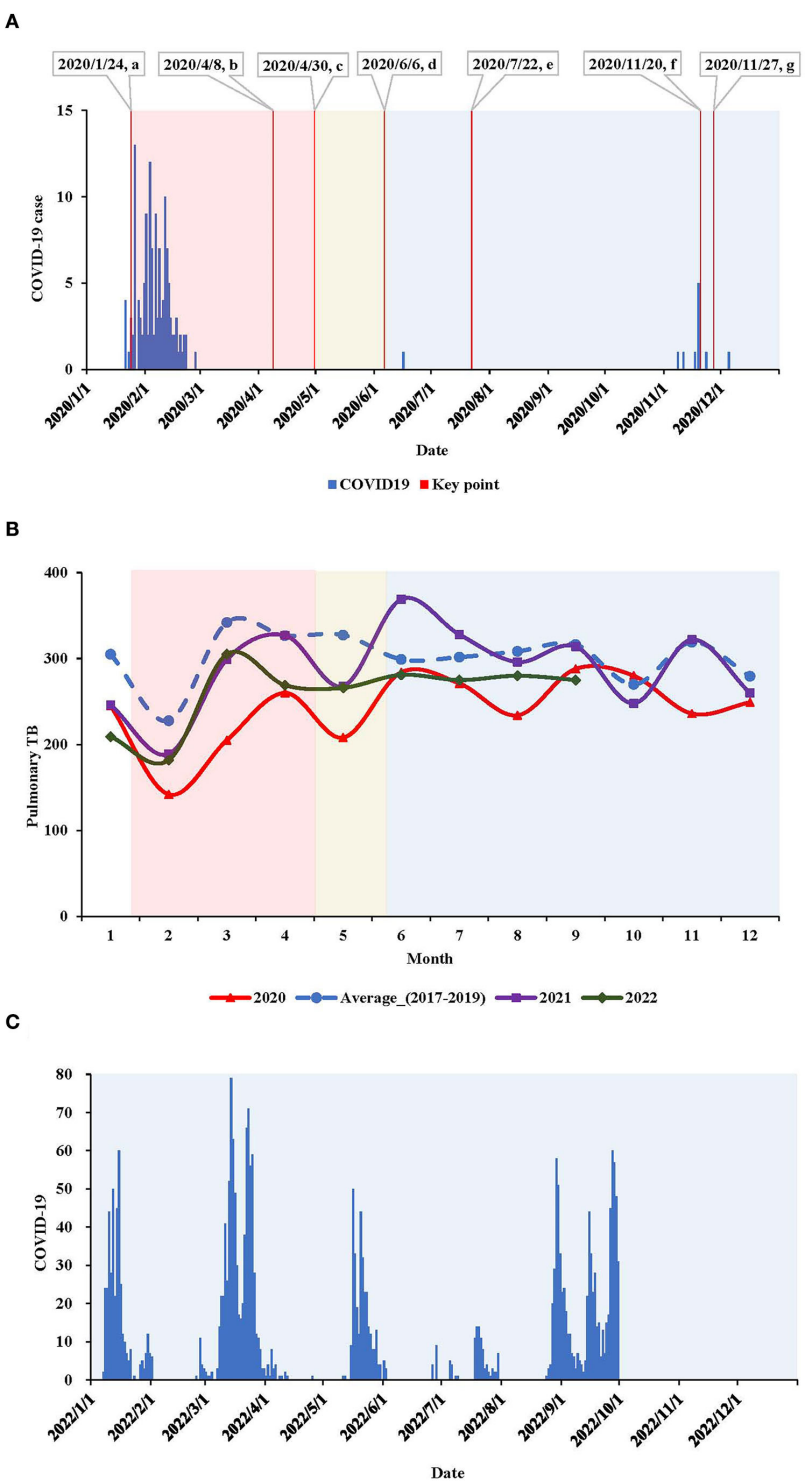
**(A)** Epidemic curve of locally transmitted COVID-19 cases and key time points in Tianjin, China, 2020. a: 2020/1/24, the first-level response was initiated; intensive lockdown being implemented, such as community constraint and restriction of public traffic; the municipal TB designated hospital was repurposed to the COVID-19 hospital and suspended TB service. b: 2020/4/8, reopen of Wuhan city where the COVID-19 was firstly learnt. c: 2020/4/30, the response to COVID-19 was downgraded to second-level; when all sectors being gradually reopening. d: 2020/6/6, the response to COVID-19 was further downgraded to the third-level, when all sectors back to normalization under basic measures for COVID-19 control, such as mask wearing in public and COVID-19 testing with presumable symptoms. e: 2020/7/22, the municipal TB designated hospital gradually restored TB healthcare, meanwhile still served as the COVID-19 hospital (for imported COVID-19 cases). f: 2020/11/20, the municipal TB designated hospital again suspended TB healthcare. g: 2020/11/27, another hospital with specialty of infectious diseases was newly appointed as an interim TB designated hospital to complement the deficiency of TB service. 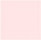 First-level response to COVID-19. 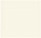 Second-level response to COVID-19. 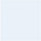 Third-level response to COVID-19. **(B)** Pulmonary TB report by month in Tianjin, China, in 2020, 2021 and 2022, compared with the average level in 2017–2019. **(C)** Epidemic curve of locally transmitted COVID-19 cases in Tianjin, China, in the three quarters in 2022.

In this study, the incidence of COVID-19 was acquired from publicly available data (http://wsjk.tj.gov.cn/; http://www.tjyun.com/). In 2020, we differentiated four periods in terms of public health emergency response to COVID-19 according to the government's announcement in Tianjin, China (Figure 2A): pre-response (before 24 January 2020: no public health intervention), first-level response (24 January−29 March 2020: strict lockdowns being implemented, such as community constraint and restriction of public traffic), second-level response (30 March 30–5 June 2020: public facilities being gradually reopened), and third-level response (after 6 June 2020: all public sectors back to normalization under basic measures for COVID-19 control, such as wearing masks in public and COVID-19 testing with presumable symptoms).

### Data analysis

We compared proportions of the postponement among patients in different categories using conventional contingency tables and tested statistical significance by χ^2^ test. A logistic regression was used to analyze factors associated with the postponement of first healthcare seeking: variables of patient characteristics were introduced as independents one by one, to calculate odds ratios (OR) and 95% confidence intervals (CI); and then variables with *P* <0.15 in the χ^2^ test were included using a backward method to calculate adjusted ORs (aOR) and 95% CIs.

We calculated the time for healthcare seeking (the days from the onset of TB symptoms to the first healthcare seeking), the time for TB diagnosis (the days from the first healthcare seeking to TB diagnosis), and the time for sputum test at the 2^nd^ month of the treatment (the days from TB diagnosis to the sputum test for the 2^nd^ month) for patients reported in the surveillance system. In each period relating to the COVID-19 response, we compared differences in characteristics between patients reported in 2020 with the patients in 2017–2019. Statistical significances were evaluated using the Wilcoxon test for median values, using the χ^2^ test for proportions.

All analyses were carried out using SAS 9.4 (SAS Institute Inc., http://support.sas.com), α = 0.05.

## Results

### Patient participants

A total of 518 pulmonary TB patients participated in the anonymous survey, including 160 (31%) diagnosed before the COVID-19 response and 358 (69%) diagnosed during the COVID-19 response. Male participants accounted for the majority (*n* = 308; 60%); age ranged from 15 to 90 years, with a median of 37 years (IQR: 28, 56). The majority were new patients who were not previously treated for TB (85%). Permanent local residents accounted for the majority (77.0%), where most of them currently live in the 16 districts in Tianjin, while 24 (5%) came from adjacent cities specifically for TB services.

### Postponement of healthcare seeking for TB

Among those 358 patients diagnosed with TB during the COVID-19 response, 61 (17%) reported postponement of their first-time healthcare seeking specifically due to COVID-19; of which, 35 (57%) postponed for ≥30 days, 8 (13%) for 14–29 days, 7 (12%) for 7–13 days, and 11 (18%) for <7 days. Reasons for postponement (multi-choice) were as follows: 39 (64%) cited fear, five (8%) cited lockdown measures (e.g., community containment, traffic restriction), nine (14.8%) cited insufficient TB service (e.g., difficulty to register for healthcare), and 12 (20%) cited screening for COVID-19.

We analyzed factors associated with the postponement due to COVID-19 (Table 2). Sex, age, residency, education, and travel time to TB clinics were not statistically associated with the postponement (*P* > 0.05). Patients who were previously treated for TB (*P* = 0.025), with symptoms of cough or fever (*P* = 0.050), and diagnosed with TB during the first-level response (*P* = 0.003) had a higher proportion of postponement than the others. In the multivariate logistic regression, the female sex (aOR = 2.0, 95% CI: 1.1–3.7), patients with previous TB treatment (aOR = 3.2, 95% CI: 1.4–7.6), and diagnosed TB during the first-level response (aOR = 3.2, 95% CI: 1.7–6.2) were risk groups to have postponement due to the COVID-19.

**Table 2:** Postponement of healthcare seeking specifically due to the COVID-19 among patients diagnosed pulmonary TB during the COVID-19 response in 2020, Tianjin, China (*n* = 358).

	**Postponement of** **healthcare seeking (%)**			**Univariate**		**Multivariate**	
	**No**	**Yes**	**Total**	***OR* (95% CI)**	***P* by chisq**	***aOR* (95% CI)**	***P* by** ** Wald-chisq**
**Gender**							
Male	189 (85.9)	31 (14.1)	220	ref	0.061	ref	0.017
Female	108 (78.3)	30 (21.7)	138	1.7 (1.0–3.0)		2.0 (1.1–3.7)	
**Age**							
<25	46 (83.6)	9 (16.4)	55	ref	0.744	NA	NA
25–44	140 (82.4)	30 (17.6)	170	1.1 (0.5–2.5)		NA	
45–64	74 (86.0)	12 (14.0)	86	0.8 (0.3–2.1)		NA	
≥65	37 (78.7)	10 (21.3)	47	1.4 (0.5–3.8)		NA	
**Residency**							
Local	232 (82.6)	49 (17.4)	281	ref	0.702	NA	NA
Migrant	65 (84.4)	12 (15.6)	77	0.9 (0.4–1.7)		NA	
**Education**							
Primary	34 (87.2)	5 (12.8)	39	ref	0.079	ref	0.134
Middle school	71 (88.8)	9 (11.3)	80	0.9 (0.3–2.8)		0.7 (0.2–2.3)	
High school	79 (85.9)	13 (14.1)	92	1.1 (0.4–3.4)		1.1 (0.3–3.4)	
College	113 (76.9)	34 (23.1)	147	2 (0.7–5.6)		1.7 (0.6–5.1)	
**Previous anti-TB treatment**							
No	275 (84.4)	51 (15.6)	326	ref	0.025	ref	0.007
Yes	22 (68.8)	10 (31.3)	32	2.5 (1.1–5.5)		3.2 (1.4–7.6)	
**Cough or fever**							
No	143 (87.2)	21 (12.8)	164	ref	0.050	ref	0.061
Yes	154 (79.4)	40 (20.6)	194	1.8 (1–3.1)		1.8 (1.0–3.2)	
**Travel time to TB clinic**							
<0.5 h	109 (83.2)	22 (16.8)	131	ref	0.808	NA	NA
0.5–1 h	115 (81.6)	26 (18.4)	141	1.1 (0.6–2.1)		NA	
≥1 h	73 (84.9)	13 (15.1)	86	0.9 (0.4–1.9)		NA	
**Period of diagnosis**							
3rd-level response	168 (88.0)	23 (12.0)	191	ref	0.003	ref	0.002
1st-level response	62 (71.3)	25 (28.7)	87	2.9 (1.6–5.6)		3.2 (1.7–6.2)	
2nd-level response	67 (83.8)	13 (16.3)	80	1.4 (0.7–3)		1.5 (0.7–3.1)	

### Supervision of anti-TB treatment

Among all 518 patients, 175 (34%) reported antituberculosis treatment without supervision during the COVID-19 response, and in the remaining 343 (66%), 187 (36%) were under supervision by medical staff or volunteers (e.g., family members), while 156 (30%) used electric pill-box or mobile phone application for medication reminder. In terms of treatment tracer, 197 (38%) received no communication from primary healthcare staff during the COVID-19 response, and among the other 321 (62%): 62 (12%) received at least one home visit, 259 (50%) received at least one phone call or online communication. Although most of the patients were prescribed anti-TB drugs from TB clinics, 30 (6%) purchased anti-TB drugs through irregular ways during the COVID-19 response, including 17 (3%) from pharmacy stores and 16 (3%) *via* online surrogates. Acquisition of antituberculosis drugs outside TB clinics accounted for 16% (9/58) of patients with known drug-resistant TB, a rate higher than 4.6% (21/460) of patients without drug-resistant TB (*P* < 0.001).

In total, 32 (6%) patients reported interruption in treatment during the COVID-19 response; including 12 (38%) who interrupted for ≥30 days, 8 (25%) for 14–29 days, and 12 (38%) for <14 days. A total of 57 patients (11%) postponed a regular visit to the TB clinic during treatment due to COVID-19, including 25 (44%) who postponed for ≥30 days, 13 (23%) for 14–29 days, and 19 (33.3 %) for <14 days. The primary reasons for the postponement included fear of COVID-19 by 34 (60%), lockdown measures by 12 (21.1%), insufficient TB service by 16 (28.1%), and being kept out of the city by 5 (8.8%).

### Finding TB cases and healthcare in hospitals

We surveyed 45 non-TB hospitals that reported presumable patients with TB, among which 27 (60%) had fever clinics (fever clinics are specifically set during the COVID-19 response in selective hospitals with the qualified ability for infection control; people seeking healthcare and with presumable COVID-19 symptoms such as fever and cough must first undergo COVID-19 screening in fever clinics). During the first-level response, 25 (56%) hospitals reported notably less presumable patients with TB, mainly due to a sharp reduction of outpatients, without fever clinics and suspension of health check-ups by their administration; however, other six (13.3%) hospitals, all of which had fever clinics, reported even more presumable patients with TB during the first-level response, mainly attributed to fever clinics and more frequent medical examinations such as CT scan (to rule out COVID-19). Notably, 9 (20%) hospitals reported referral delays of presumable patients with TB to TB clinics, of which eight (8/9) cited the shutdown of the municipal TB designated hospital as the reason.

Among the 12 TB clinics or designated hospitals in the city, two (2/12) had ever suspended TB healthcare service during the first-level response, including the municipal TB designated hospital that was repurposed to the COVID-19 designated hospital. Seven (7/12) of them had ever reassigned medical staff of TB to work for COVID-19. Although there was no shortage of TB medicine or reagent, insufficiency of medical staff and equipment for TB diagnosis was the main complaint by the TB clinics during the COVID-19 response.

### Pulmonary TB surveillance

In 2020, the pulmonary TB report declined by 19.6% compared with the average level in 2017–2019 in Tianjin, China (Table 3, Figure 2). The decline was, respectively, 36.8% during the first-level response, 23.5% during the second-level response, and 13.3% during the third-level response. The pulmonary TB report was correlated with the COVID-19 epidemic, response intensity, and TB healthcare accessibility, and the sharpest decline occurred during the first wave of the COVID-19 epidemic. In 2021, when no locally transmitted COVID-19 cases emerged, and a hospital with a specialty in infectious diseases was newly appointed as an interim TB designated hospital to complement the insufficiency of TB health care (the former one was repurposed as a COVID-19 hospital), thus pulmonary TB report was restored; however, it was still 4.3% down from the average level reported in 2017–2019. When locally transmitted COVID-19 resurged in the first quarter of 2022, the pulmonary TB report again declined by 20.4% compared to 2017–2019.

**Table 3:** Comparison among patients with pulmonary TB reported during different time periods in 2020, and the same time intervals in 2017–2019 and 2021, Tianjin, China.

	**2017–2019 y**	**2020 y**	**2021 y**	***P***
Pulmonary TB report (cases)^a^				
Pre-response	231	239	172	NA
1st-level	962	608	874	
2nd-level	374	286	356	
3rd-level	2,056	1,769	2,064	
Whole year	3,622	2,902	3,466	
Time for healthcare seeking (days)^b^			
Pre-response	14 (2, 33)	12 (3, 30)	14.5 (4, 47.5)	0.137
1st-level	14 (3, 35)	11 (2, 31)	14 (3, 39)	0.032
2nd-level	13 (1, 31)	14 (2, 51)	13 (1, 36)	0.234
3rd-level	12 (1, 31)	11 (2, 39)	11 (1, 32)	0.035
Whole year	13 (2, 31)	12 (2, 36)	12 (1, 34)	0.586
Time for TB diagnosis (days)^c^				
Pre-response	13 (5, 25)	7 (4, 17)	9 (4, 24)	0.007
1st-level	11 (4, 21)	7 (3, 15)	8 (3, 18)	<0.001
2nd-level	12 (6, 24)	8 (3, 18)	9 (4, 24)	<0.001
3rd-level	12 (5, 22)	13 (6, 26)	7 (3, 20)	<0.001
Whole year	12 (5, 22)	11 (5, 22)	8 (3, 20)	<0.001
Time for sputum test at 2^nd^ month (days) ^d^			
Pre-response	61 (51, 71)	66 (59, 81)	63 (52, 71)	<0.001
1st-level	60 (50, 70)	65 (59, 74)	63 (53, 73)	<0.001
2nd-level	61 (49, 70)	63 (57, 71)	62 (53, 74)	0.015
3rd-level	61 (50, 70)	62 (49, 71)	60 (47, 69)	<0.001
Whole year	61 (50, 70)	63 (54, 72)	61 (50, 71)	<0.001
Proportion of sputum test at 2^nd^ month [% (*n*)]			
Pre-response	89.3 (619/693)	82.0 (196/239)	92.4 (159/172)	0.002
1st-level	88.3 (2,548/2,885)	90.3 (549/608)	92.3 (807/874)	0.003
2nd-level	87.5 (981/1,121)	94.4 (270/286)	89.9 (320/356)	0.003
3rd-level	86.9 (5,357/6,168)	89.0 (1,575/1,769)	86.3 (1,782/2,064)	0.026
Whole year	87.5 (9,505/10,867)	89.3 (2,590/2,902)	88.5 (3,068/3,466)	0.018
Coverage of Xpert test [% (*n*), pleurisy not included]			
Pre-response	49.2 (298/606)	78.6 (165/210)	94.5 (154/163)	<0.001
1st-level	55.3 (1,429/2,586)	91.6 (531/580)	94.7 (790/834)	<0.001
2nd-level	60.9 (612/1,005)	97.1 (263/271)	88.2 (305/346)	<0.001
3rd-level	56.9 (3,166/5,569)	94.6 (1,596/1,688)	88.6 (1,755/1,980)	<0.001
Whole year	56.4 (5,505/9,766)	92.9 (2,555/2,749)	90.4 (3,004/3,323)	<0.001
Bacteriological confirmation [% (n), pleurisy not included]			
Pre-response	58.6 (355/606)	64.8 (136/210)	63.8 (104/163)	0.197
1st-level	67.2 (1,737/2,586)	61.6 (357/580)	68.8 (574/834)	0.012
2nd-level	67.4 (677/1,005)	62.4 (169/271)	61.0 (211/346)	0.122
3rd-level	68.5 (3,812/5,569)	65.9 (1,112/1,688)	64.3 (1,274/1,980)	<0.001
Whole year	67.4 (6,581/9,766)	64.5 (1,774/2,749)	65.1 (2,163/3,323)	<0.001
Treatment success among non-MDR/RR-TB [% (n)]^e^			
Pre-response	95.1 (619/651)	94.3 (216/229)	93.8 (137/146)	0.789
1st-level	94.4 (2,573/2,727)	94.9 (541/570)	94.3 (591/627)	0.854
2nd-level	93.8 (993/1,059)	95.2 (258/271)	95.4 (188/197)	0.494
3rd-level	93.7 (5,382/5,742)	94.0 (1,574/1,674)	93.1 (135/145)	0.856
Whole year	94.0 (9,567/10,179)	94.4 (2,589/2,744)	94.3 (1,051/1,115)	0.747

a The number in 2017–2019 was the average value during the 3 years;

b time interval between onset of TB symptoms and the first healthcare seeking in a TB clinic;

c time interval between first healthcare seeking in a TB clinic and TB diagnosis;

d time interval from TB diagnosis to sputum test at the 2^nd^month of treatment;

e The rate was calculated for patients without MDR/RR-TB (defined as resistance to Rifampicin, with or without resistance to Isoniazid), and in 2021, the rate was calculated for patients reported between January and June.

Pulmonary TB cases reported during the first-level response in 2020 sought timely healthcare compared to that in 2017–2019 (*P* = 0.032). The time for TB diagnosis was shorter in 2020 and 2021 compared to that in 2017–2019 (*P* < 0.001), which was 4–6 days sooner during the pre-response, first-level, and second-level response in 2020. The proportion of the sputum test at the 2^nd^ month of treatment was 82.0% for patients with TB reported in the prophase of COVID-19, lower than 89.3% in 2017–2018 and 92.4% in 2021 (*P* = 0.002). The time for sputum test in the 2^nd^ month was longer among patients diagnosed with TB in 2020 compared to 2017–2019 and 2021 (*P* < 0.001), especially among patients reported in COVID-19 prophase and first-level response in 2020, which was 5 days later than in 2017–2019. Although the coverage of Xpert^®^ MTB/RIF assay (Xpert) increased from 56.4% in 2017–2019 to 92.9% in 2020 and 90.4% in 2021, the proportion of bacteriological confirmation decreased from 67.4% in 2017–2019 to 64.5% in 2020 and persistently lower as 65.1% in 2021 (*P* < 0.001). Among patients who had completed the course of TB treatment, the proportions of treatment success were over 94% in the years 2020 and 2021, which were not statistically different compared to 2017–2019 (*P* = 0.747).

## Discussion

The COVID-19 pandemic caused stress on all aspects of public health globally (4). In this study, through a pre-planned survey combined with surveillance, we learned the overall impact of COVID-19 on TB prevention and control in Tianjin, China. Although there were only two waves of locally transmitted COVID-19 epidemics with <150 total incident COVID-19 cases in 2020, pulmonary TB report reduced by 20% in Tianjin, a proportion that was higher than the 18% reductions in TB notifications worldwide during 2019–2020 (11). The sharpest decline occurred in February, similar to other studies conducted in China (14–17). Over the previous years, there was a regular decline in pulmonary TB reported around February due to the national holiday for the Chinese Lunar New Year (14–17). However, in 2020, this regularity was overlaid by the COVID-19 epidemic, when a comprehensive package of public health interventions was in place. As COVID-19 was mitigated, the TB report gradually rebounded, suggesting that the declined report of pulmonary TB was correlated with the number of COVID-19 cases, the COVID-19 response level, and availability of TB services.

Public health interventions had played a great role in the successful control of COVID-19 (2, 3, 19). These measures included social distancing and community containment; however, it has been postulated to facilitate *M. tuberculosis* transmission within households (20, 21). Because, approximately, one-third of the population in the world is estimated to have latent TB infection, potentially with protracted and uncertain latency toward progression to TB disease (22), it is not plausible that the real TB incidence could decline in such a short term. As demonstrated in 2021, when no locally transmitted COVID-19 cases emerged and TB services resumed in Tianjin, the report of pulmonary TB was restored. The universal decline of outpatients in non-TB hospitals, and the postponement of healthcare seeking due to COVID-19 among patients with TB, indicated that hesitation and hindrance for accessing service might lead to the decline of TB cases reported.

Postponement for healthcare seeking caused a delay in TB diagnosis, not only exacerbated symptomology and clinical disease but also increased the risk of transmission (20, 21, 23). Our survey found that the fear of COVID-19 was the most important reason for patients' postponement of healthcare seeking, especially during the first-level response (*aOR* = 3.2, 1.7–6.2), which echoed the sharpest decline of pulmonary TB reported during this period. Both TB and COVID-19 could be stigmatized conditions (12), causing hesitation in healthcare seeking (24, 25). In addition, the female population and patients with previous TB treatment had a higher risk for postponement. Several surveys suggested that women were more likely to comply with non-pharmaceutical interventions and to consider COVID-19 as a real risk (4). Patients with previous anti-TB treatment were risk group of drug resistance (26), and their postponement potentially increased the spread of drug resistance. The results suggest interventions to encourage healthcare seeking behavior, promote confidentiality, and prevent discrimination during the response to a public health emergency.

Besides fear, public health interventions caused inconvenience to healthcare (13, 15–17). In our study, the accessibility of TB service became the major concern due to the repurposing of the municipal TB designated hospital. It is worth noting that, at the end of 2020, another hospital with a specialty in infectious diseases was newly appointed as an interim TB designated hospital to complement the insufficiency of TB service. Nevertheless, when a new wave of COVID-19 resurged in the first quarter of 2022, a 20% decline in pulmonary TB report still occurred. The fact suggests a persistent impact of COVID-19 on TB control by factors more than TB healthcare accessibility.

Delay in diagnosis and treatment for TB was estimated to be the greatest concern leading to an increase in death (10). A study in Ningxia, China, reported a longer delay of healthcare seeking among patients diagnosed with TB during the intensive period of COVID-19, resulting in a higher proportion of cavitation and smear-positivity (17). Contrary to that, in our surveillance, compared with in 2017–2019, patients reported during the first-level COVID-19 response had sought healthcare even timelier; and meanwhile the proportion of bacteriological confirmation for the patients decreased, although the coverage of Xpert testing remained high (27). The result seemed paradoxical to the questionnaire survey that a significant proportion of patients postponed their healthcare seeking due to COVID-19. The paradox may be influenced by two aspects, on the one hand, the COVID-19 epidemic impeded healthcare seeking either due to fear or public interventions causing postponement; on the other hand, patients' awareness of respiratory symptoms was improved and demanded timely healthcare during the COVID-19 pandemic.

As an intervention to intensify COVID-19 detection, patients with presumable symptoms such as cough and fever were required to first undergo COVID-19 screening in fever clinics, which were specifically established in appointed hospitals. Since pulmonary TB may manifest similar symptoms as COVID-19, the fever clinics provide both challenges and opportunities for finding TB cases (18). In our survey, some participants reported postponement of healthcare seeking for TB due to COVID-19 screening, calling for the efficient referral of TB in fever clinics. On the other hand, medical examinations such as CT scans were carried out more frequently for COVID-19 screening in fever clinics, which also helped to find TB cases. In countries with high TB burden, synchronous dual case finding of COVID-19 and TB was recommended (28). The fever clinic can be a way to dual case findings of COVID-19 and TB as indicated by our study.

Insufficient treatment support and medication stockouts may hamper treatment for patients with TB during the COVID-19 epidemic (21). In our survey, a part of the patients postponed regular visits to TB clinics and interrupted anti-TB treatment. Treatment interruption was the most important factor in treatment failure and acquired drug resistance (29–31). Moreover, anti-TB drugs obtained from pharmacies or online surrogates instead of prescriptions from TB clinics, brought more safety concerns, especially for patients requiring second-line anti-TB drugs. Apart from anti-TB drugs, insufficient supervision also resulted in the discontinuation of anti-TB treatment. The absence of supervision might relate to the shortage of healthcare staff when they were reallocated to COVID-19 control (12, 24). In this context, there is a good opportunity to uptake digital tools for TB management, such as video directly observed therapy for adherence interventions (11, 12, 28, 32).

Due to the long duration of anti-TB treatment, limited data had revealed the impact of COVID-19 on TB treatment outcomes (21). In Brescia, Italy, during the early 2 months of the COVID-19 epidemic, the proportion of patients with TB lost to follow-up was significantly higher compared to the previous year (10.8 vs. 2.6%, *P* = 0.03), and more TB death occurred (4.6 vs. 0%, *P* = 0.04) (33). Globally, there was an estimation of 0.1 million TB deaths increase in 2020 compared to 2019 because of reduced access to the diagnosis and treatment of TB in the face of the COVID-19 pandemic (11). In our study, although there was a disruption in TB service, the treatment success for patients reported since 2020 was non-inferior to the previous years, in accordance with a similar study conducted in Ningxia, China (17). The non-inferior treatment outcome benefited from the successful control of COVID-19, allowing gradual recovery of the disrupted TB service. However, in the case of COVID-19 resurgence or other public health emergencies, early preparation is essential, such as the adoption of digital tools for TB management.

There are some limitations to this study. Participants in the anonymous survey were conveniently sampled among patients who came to TB clinics; however, probable patients bearing TB symptoms without healthcare seeking could not be investigated. The selection bias might underestimate the impact of COVID-19 on TB diagnosis and treatment. The long-term impact of COVID-19 on TB requires further investigation (18, 23).

## Conclusion

Both reduction of patients' healthcare seeking and the shrinkage of TB services lead to the decline of TB reported during the COVID-19 response. We suggest the following remedial actions: (1) an integrated program for dual case-finding of COVID-19 and TB; (2) interventions to mitigate fear and stigma to facilitate presumable patients in healthcare seeking; (3) guarantee of essential treatment and supervision for TB using digital strategies.

## Data availability statement

The raw data supporting the conclusions of this article will be made available by the authors, without undue reservation.

## Ethics statement

Ethical review and approval was not required for the study on human participants in accordance with the local legislation and institutional requirements. Written informed consent to participate in this study was provided by the participants' legal guardian/next of kin.

## Author contributions

GZ conceived and designed the study, analyzed the data, and wrote the manuscript. YY, WZ, JS, SC, and XP carried out the fieldwork and collected the data. JO and PM provided support for data analysis and proofread the manuscript. MC and FZ provided support for the survey and interpreted the results. All authors were involved in manuscript revision and approved the final manuscript.

## Funding

The research was sponsored by Tianjin Health Research Project (grant number: ZC20031).

## Conflict of interest

The authors declare that the research was conducted in the absence of any commercial or financial relationships that could be construed as a potential conflict of interest.

## Publisher's note

All claims expressed in this article are solely those of the authors and do not necessarily represent those of their affiliated organizations, or those of the publisher, the editors and the reviewers. Any product that may be evaluated in this article, or claim that may be made by its manufacturer, is not guaranteed or endorsed by the publisher.

## Abbreviations

IQR: interquartile range
TB: tuberculosis
Xpert: Xpert^®^ MTB/RIF assay
TBIMS: TB Information Management System.
